# Transcriptome analysis provides insights into light condition effect on paclitaxel biosynthesis in yew saplings

**DOI:** 10.1186/s12870-022-03958-2

**Published:** 2022-12-12

**Authors:** Taotao Li, Bingbing Li, Chunli Liao, Huamin Zhang, Lianzhe Wang, Taotao Fu, Shouyu Xue, Tao Sun, Xiaolan Xu, Xin Fan, Le Li, Genglin Liu, Fengling Yang, Xuan Ma

**Affiliations:** 1 College of Life Sciences and Engineering, Henan University of Urban Construction, Pingdingshan, 467036 Henan China; 2 National Key Laboratory of Crop Genetic Improvement, Huazhong Agricultural University, Wuhan, 430070 China

**Keywords:** *Taxus chinensis*, High light, Transcriptome, Paclitaxel biosynthesis, Photosynthetic system

## Abstract

**Background:**

*Taxus* is a rare gymnosperm plant that is the sole producer of the anticancer drug paclitaxel. The growth and development of *Taxus* is affected by environmental factors such as light. However, little is known about how light conditions affect growth and metabolic processes, especially paclitaxel biosynthesis.

**Results:**

In this study, we applied three different light conditions to *Taxus chinensis* young saplings and investigated the physiological response and gene expression. Our observations showed that exposure to high light led to oxidative stress, caused photoinhibition, and damaged the photosynthetic systems in *T. chinensis*. The paclitaxel content in *T. chinensis* leaves was significantly decreased after the light intensity increased. Transcriptomic analysis revealed that numerous genes involved in paclitaxel biosynthesis and phenylpropanoid metabolic pathways were downregulated under high light. We also analyzed the expression of JA signaling genes, bHLH, MYB, AP2/ERF transcription factors, and the *CYP450* families that are potentially related to paclitaxel biosynthesis. We found that several *CYP450s, MYB* and *AP2/ERF* genes were induced by high light. These genes may play an important role in tolerance to excessive light or heat stress in *T. chinensis*.

**Conclusions:**

Our study elucidates the molecular mechanism of the effects of light conditions on the growth and development of *T. chinensis* and paclitaxel biosynthesis, thus facilitating the artificial regeneration of *Taxus* and enhancing paclitaxel production.

**Supplementary Information:**

The online version contains supplementary material available at 10.1186/s12870-022-03958-2.

## Background

Light plays a major role in plant growth and development. As the energy source, light is absorbed by plants, converted into chemical energy, and stored in the formed organic compounds through photosynthesis. Light is also a developmental signal that facilitates photomorphogenesis in plants [1]. Its quality and quantity under natural conditions largely affect plant growth and development. For example, a lack of light can hinder photomorphogenesis and plant growth, causing leaf etiolation and survival descent [2]. When exposed to excessive or high light, plants’ capacity to assimilate CO_2_ and photosynthetic efficiency will be depressed, and the photosynthetic electron transport chain will generate reactive oxygen species (ROS) and radicals, causing severe photoinhibition and oxidative stress [3–5].

Yew (*Taxus* L.) is a rare ancient woody gymnosperm that produces paclitaxel (Taxol), a well-known anticancer medicine. *Taxus* is an endangered plant due to its weak regenerative properties and slow growth. In China, four species and one variety, namely, *T. chinensis* (Pilger) Rehd., *T. wallichiana* Zucc., *T. cuspidate* Sibe. et Zucc., *T. yunnanensis*, and *T. chinensis* var. *mairei*, are found in *Taxeae* [6]. *Taxus* generally grows in a mild environment with high humidity, moderate temperature and fertilized land. It is one of the most shade-tolerant trees although it grows well under full sunlight [7, 8]. In comparison with adult trees, young *Taxus* saplings grow in forested landscapes shaded with dense canopies. Several studies have suggested that *Taxus* plants and other tree species respond to light conditions depending on age or developmental stage [9, 10]. However, studies have yet to clarify how light conditions influence young *Taxus* sapling growth, morphogenesis, and physiological responses.

Paclitaxel biosynthesis in *Taxus* is a complex metabolic pathway that requires at least 19 enzymatic steps and more than 20 enzymes [11, 12]. To date, this pathway has 13 identified enzymes, including one taxadiene synthase (TS), which cyclizes the diterpenoid precursor geranylgeranyl diphosphate (GGPP) into taxadiene [13]; five cytochrome P450 (CYP450) hydroxylases (T5αH, T10βH, T2αH, T7βH, and T13αH) [14–16] and five acyltransferases (TAT, TBT, DBAT, BAPT, and DBTNBT) [17–19], which decorated the taxane skeleton to form the final product paclitaxel; and two enzymes (PAM and T2′OH) in the β-phenylalanoyl side chain [20]. With the application of next-generation sequencing (NGS) technology to *Taxus*, valuable genetic information can be obtained to elucidate the molecular basis of the paclitaxel biosynthesis pathway and identify key enzymes involved in the pathway. Several studies based on transcriptomic analysis have identified some potential candidate genes and transcription factors that are possibly involved in the paclitaxel biosynthetic pathway [21, 22]. Chromosome-level genomes of three *Taxus* species (*T. chinensis* var. *mairei*, *T. wallichiana*, and *T. yunnanensis*) have been published recently [23–25], thus greatly advancing research on paclitaxel biosynthesis. However, information about how environmental conditions affect paclitaxel biosynthesis and metabolic processes in *Taxus* is limited.

In the present study, we analyzed the physiological and transcriptomic responses of *T. chinensis* to different light conditions that simulate the light intensity in *Taxus* natural habitat. We investigated gene transcription in the paclitaxel biosynthetic pathway by transcriptome sequencing. Our research revealed that high light affects growth and gene expression in young *T. chinensis* plants and provides insights into the paclitaxel biosynthesis pathway.

## Results

### Effects of high light on the photosynthesis system of T. chinensis

**Fig. 1:**
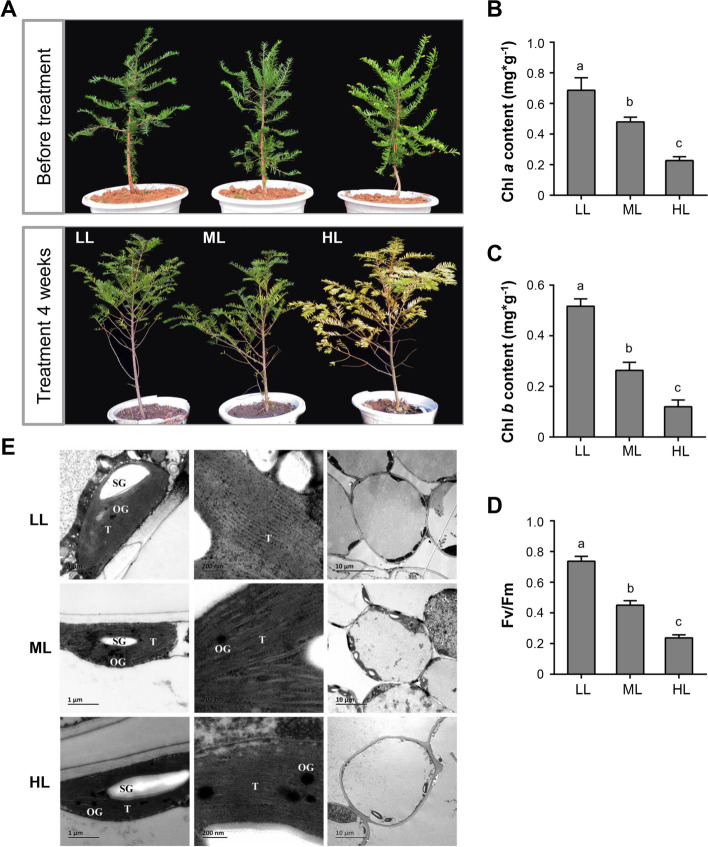
Effects of different light conditions on the photosynthesis system of *T. chinensis*. **A** Phenotype of young *T. chinensis* plants under different light intensity (high light [HL, 13,000 lx], medium light [ML, 10,000 lx], and low light [LL, 7,000 lx]) treatments. **B** The content of chlorophyll *a* and **C** chlorophyll *b* in leaves of *T. chinensis* under different light intensity treatments. Data shown are the average mean ± *SE* of three replicates (*n* = 3). Different letters above the bars indicate statistical significance at *P* < 0.05 level among different treatment groups according to Tukey’s test. **D** The maximum quantum yield (Fv/Fm) in leaves of *T. chinensis* under different light intensity treatments. Data shown are the average mean ± *SE* of three replicates (*n* = 3). **E** Ultrastructure of *T. chinensis* chloroplasts of different light intensity treatments. T, OG, and SG indicate the thylakoid lamellae, osmiophilic globule, and starch grain, respectively. Scale (1 μm, 0.2 μm, and 10 μm)

To investigate the effect of different light intensities on young *T. chinensis* plants, we applied three light conditions (high light [HL, full sunlight], illuminance 13,000 lx; medium light [ML, semi-shading condition], 10,000 lx; and low light [LL, shading condition, as control group], 7,000 lx) to 5-year-old *T. chinensis* plants. After 4 weeks, the ML and LL treatment groups had no obvious phenotypic variations. However, the *T. chinensis* leaves turned yellow in the HL group, and a severe phenotype of excessive light stress was observed (Fig. 1A). To analyze the effect of light conditions on the photosynthetic apparatus of *T. chinensis*, we measured chlorophyll content and the chlorophyll *a* (Chl *a*) fluorescence parameter (F*v*/F*m*, maximum quantum yield) and observed chloroplast ultrastructure. The Chl *a* and Chl *b* contents significantly decreased in the ML and HL treatment groups compared with the LL treatment (Fig. 1B and C). Similarly, F*v*/F*m* measurements showed that the maximum quantum yields of the plants in the ML and HL groups were significantly lower than those in the LL group (Fig. 1D). Chloroplast ultrastructure observation showed that the number of osmiophilic granules (OGs) in chloroplasts obviously increased in the ML and HL groups compared with that in the LL group. Starch granules were also enlarged in the HL group, and the number of chloroplasts was largely reduced compared with that in the LL group. This result suggested that ultrastructural organization was impaired and abnormal in the ML and HL groups (Fig. 1E). These observations showed that chloroplasts were damaged, and the chlorophyll content was significantly decreased under long-term exposure to high light conditions (ML and HL groups), thus the photosynthesis of *T. chinensis* substantially decreased.

### Effects of light conditions on the antioxidant system and paclitaxel content

**Fig. 2:**
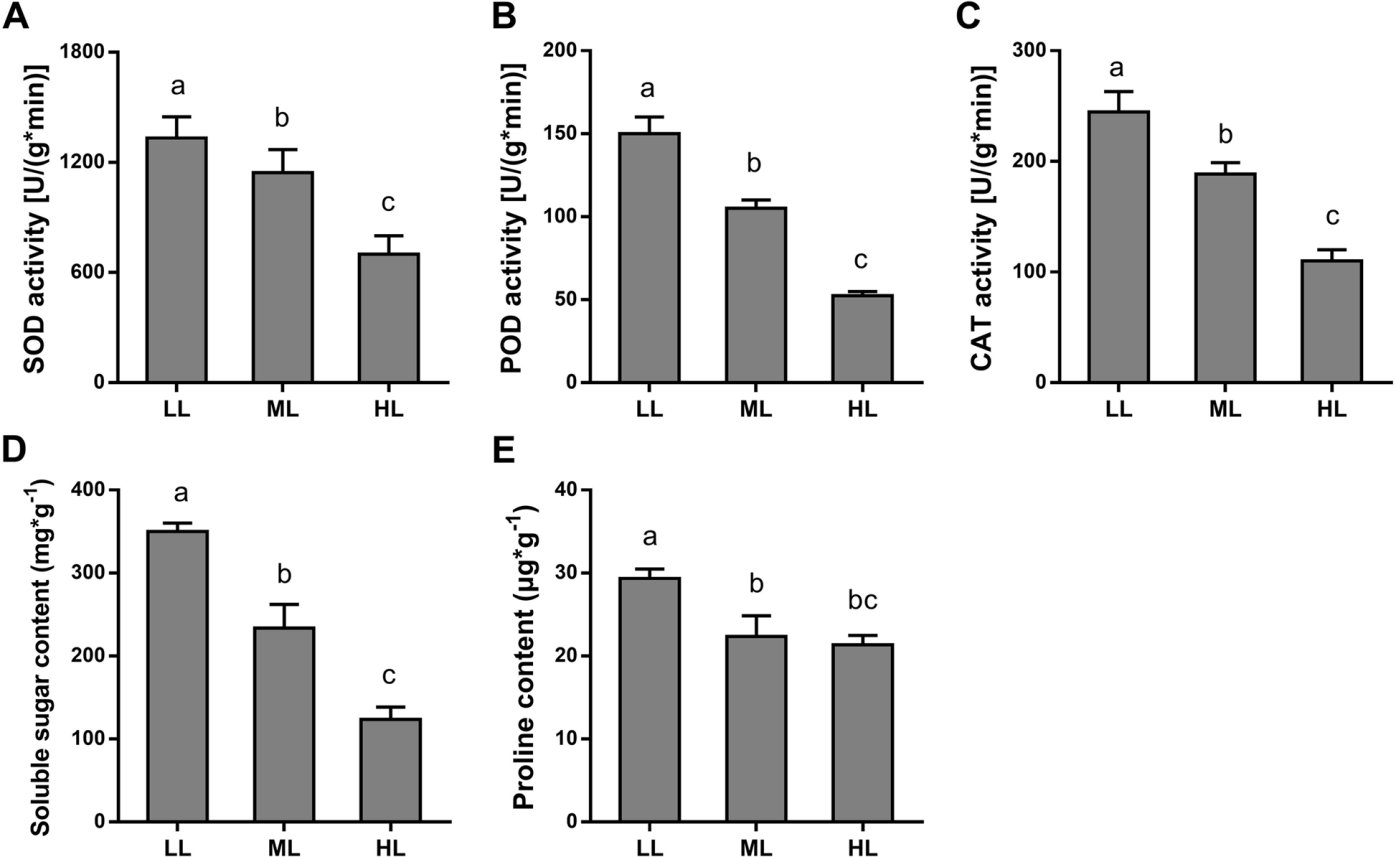
Effects of different light conditions on the antioxidant system of *T. chinensis. ***A** Superoxide dismutase (SOD), **B** Peroxidase (POD), **C** Catalase (CAT) activities in leaves of *T. chinensis* under different light intensity treatments. **D** Soluble sugar content, **E** Proline concentration in leaves of *T. chinensis* under different light intensity treatments. Data shown are the average mean ± *SE* of three replicates (*n* = 3). Different letters above the bars indicate statistical significance at *P* < 0.05 level among different treatment groups according to *Tukey’s* test

To assess the effects of the three light conditions on the antioxidant system of *T. chinensis*, we determined the activities of three antioxidant enzymes (SOD, POD, and CAT). The activities of SOD, POD, and CAT significantly decreased in the ML and HL groups compared with those in the LL group, and the lowest levels were observed in the HL group (Fig. 2A-C). Soluble sugar and proline contents were also measured in *T. chinensis* leaves in the three groups. The contents of soluble sugar and proline in the LL group were significantly higher than those in the other groups and were lowest in the HL group (Fig. 2D and E), but the proline content was not significantly different between the ML and HL groups.

**Fig. 3:**
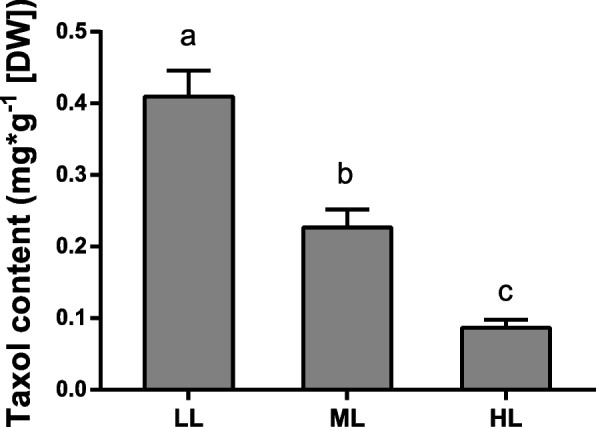
The content of Taxol in *T. chinensis* leaves under different light intensity treatments. Data shown are the average mean ± *SE* of three replicates (*n* = 3). Different letters above the bars indicate statistical significance at *P* < 0.05 level among different treatment groups according to *Tukey’s* test

We measured the taxol content in *T. chinensis* leaves through HPLC‒MS/MS and found that the taxol content in the LL group was significantly higher than that in the ML and HL groups, but the lowest content was detected in the HL group (Fig. 3). This result indicated that high light impeded paclitaxel biosynthesis in *T. chinensis*.

### Identification of differentially expressed genes (DEGs) in T. chinensis under different light intensities

**Fig. 4:**
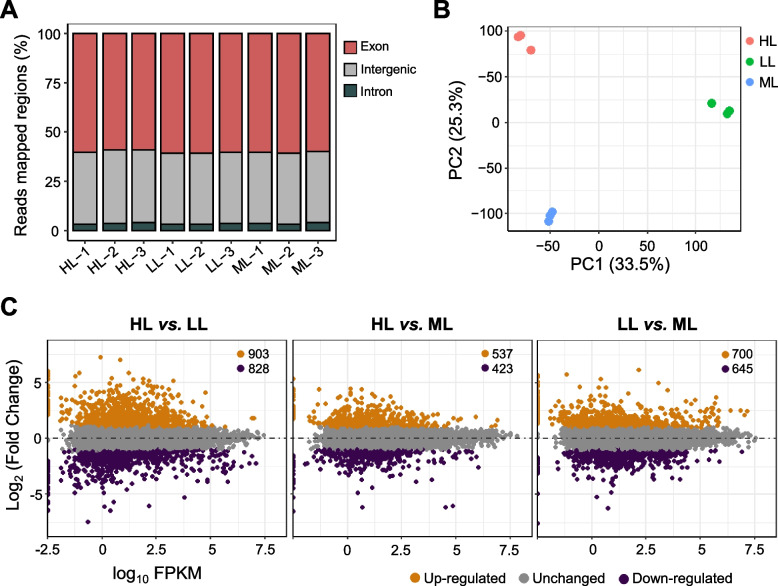
Identification of differentially expressed genes (DEGs) in *T. chinensis* under different light conditions. **A** The alignment of RNA-seq reads on the genome regions of *T. chinensis*. **B** Principal component analysis of transcriptomes of *T. chinensis* under different light intensity treatments. Red circles represent HL, green circles represent LL, and blue circles represent ML. For each treatment group, three biological replicates are shown. **C** MA-plots showing the DEGs identified in three comparisons (HL *vs.* LL, HL *vs.* ML, and LL *vs.* ML). Fold change > 2, FDR < 0.01

To study the influence of different light conditions on gene transcription in *T. chinensis*, we performed RNA sequencing (RNA-seq) of plant leaves collected from the LL, ML and HL groups (with light treatment for 4 weeks). We sequenced three biological replicates for each treatment group and obtained 68.70 Gb of data from nine cDNA libraries. After removing the low-quality reads, we obtained an average of 25.5 million clean reads for each library (Table 1). We aligned the RNA-seq reads to the *T. chinensis* reference genome, which was recently published [23]. The average total mapping rate was 85.55%, and the unique mapping rate was 80% (Table 1). Approximately 60% and 3 ~ 4% of the reads were aligned in the exon and intron regions, respectively (Fig. 4A; Supplementary Figure 2). These sequences mainly consisted of the alternative splicing (AS) of genes. The remaining 36% of the reads were aligned in the intergenic region, which might be transposon genes or regulatory element sequences. The number of transcripts (TPM > 1) detected in the transcriptome of each treatment group was comparable, and the average was 19,478 transcripts (Table 1). The transcriptomes of the three biological replicates of each treatment group were highly correlated (Pearson’s correlation coefficient of approximately 0.99–1.0) (Supplementary Figure 1). Principal component analysis (PCA) showed a distinct transcriptome feature between the three groups, and LL was distal from the HL and ML groups on PC1 (34% of the explained variance; Fig. 4B). Therefore, increasing light intensity substantially affected gene expression in young *T. chinensis* plants.

**Table 1:** Summary of alignment of *T. chinensis* RNA-seq data

Treatments	Replicates	Total reads (millions)	Total aligned reads (millions)	Uniquely aligned reads (millions)	Multiple aligned reads (millions)	Transcripts with TPM > 1
LL	1	26.48	22.75 (85.90%)	21.63 (81.69%)	1.11 (4.20%)	19,449
LL	2	25.56	22.26 (87.08%)	19.27 (75.38%)	1.16 (4.54%)	19,552
LL	3	27.26	23.47 (86.10%)	22.31 (81.85%)	1.16 (4.25%)	19,575
ML	1	22.75	19.56 (85.95%)	18.50 (81.30%)	1.06 (4.65%)	19,321
ML	2	25.82	22.17 (85.87%)	21.00 (81.21%)	1.20 (4.66%)	19,369
ML	3	26.27	25.50 (85.65%)	21.29 (81.05%)	1.21 (4.60%)	19,516
HL	1	24.29	20.59 (84.77%)	19.37 (79.73%)	1.22 (5.04%)	19,320
HL	2	24.91	21.06 (84.54%)	19.80 (79.49%)	1.26 (5.05%)	19,562
HL	3	26.14	21.98 (84.11%)	20.47 (78.30%)	1.52 (5.82%)	19,639

**Fig. 5:**
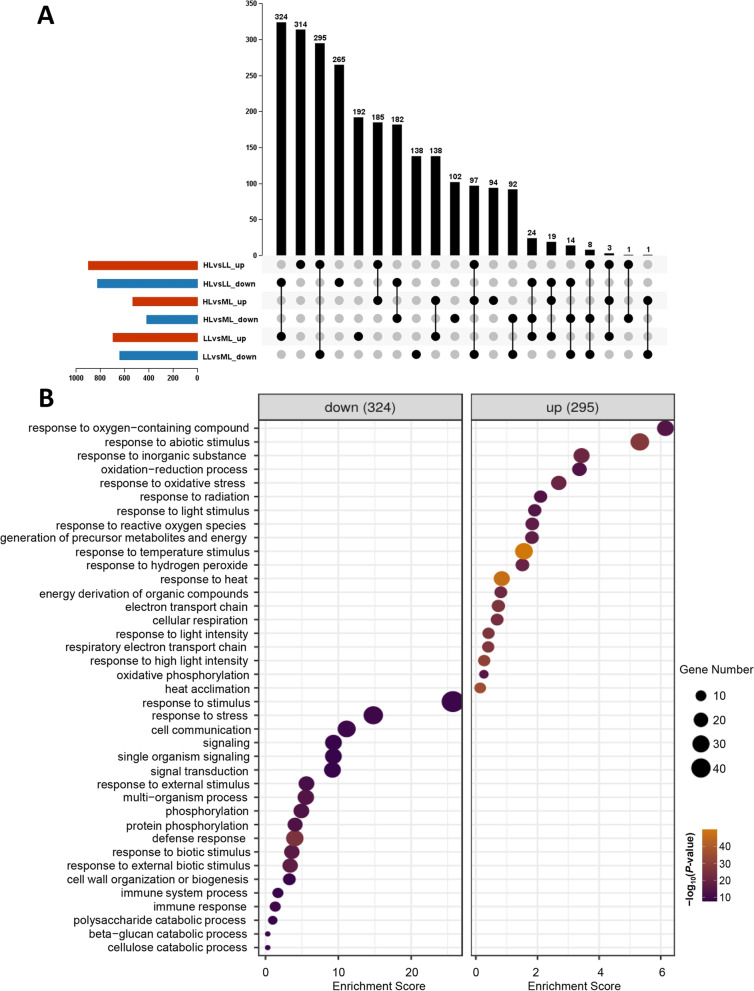
Upset-plots and GO enrichment analysis of the overlapped DEGs in *T. chinensis* under different light conditions. **A** Upset-plots of the overlapping DEGs among multiple comparisons in *T. chinensis* under different light intensity treatments. **B** GO enrichment analysis of downregulated genes (left) and upregulated genes (right) between the two comparisons of HL *vs.* LL and LL *vs.* ML

DEGs were identified in three comparisons (HL *vs.* LL, HL *vs.* ML, and LL *vs.* ML) with a fold-change cutoff (|log2FC|> 1, FDR < 0.01). A large number of DEGs were identified in HL *vs.* LL (total of 1731: 903 upregulated and 828 downregulated) and LL *vs.* ML (total of 1345: 700 upregulated and 645 downregulated). The number of DEGs in HL *vs.* ML was less than that in the two groups, with a total of 960 DEGs: 537 upregulated and 423 downregulated (Fig. 4C). We analyzed the overlapping DEGs in the three comparisons (HL *vs.* LL, HL *vs.* ML, and LL *vs.* ML). As shown in Fig. 5A, in HL *vs.* LL and HL *vs.* ML, 185 and 182 genes were commonly upregulated and downregulated, respectively. In HL *vs.* ML and LL *vs.* ML, 138 and 92 genes were commonly upregulated and downregulated, respectively. However, few DEGs in the three comparisons were commonly upregulated and downregulated. Interestingly, we found that numerous DEGs in HL *vs.* LL overlapped with those in LL *vs.* ML. Among them, 324 downregulated genes in HL *vs.* LL overlapped with the upregulated genes in LL *vs.* ML, and 295 upregulated genes in HL *vs.* LL overlapped with the downregulated genes in LL *vs.* ML (Fig. 5A).

To further determine the functional roles of the DEGs that respond to light intensity, we performed GO enrichment analysis of the overlapping DEGs between HL *vs.* LL and LL *vs.* ML and mainly verified the classifications of the biological processes involved. In HL *vs.* LL, the 324 downregulated genes were mainly enriched in several temperature- and light-responsive and oxidation–reduction processes, such as response to temperature stimulus (GO:0,009,266), response to heat (GO:0,009,408), response to high light intensity (GO:0,009,644), respiratory electron transport chain (GO:0,022,904), response to oxidative stress (GO:0,006,979), and response to radiation (GO:0,009,314) (Fig. 5B, left). The 295 upregulated genes were primarily enriched in defense response (GO:0,006,952), phosphorylation (GO:0,016,310), immune response (GO:0,006,955), and cell wall related to metabolic processes, such as cellulose catabolic process (GO:0,030,245) and cell wall organization or biogenesis (GO:0,071,554) (Fig. 5B, right). Therefore, numerous genes responding to temperature or light stimuli were highly induced by increasing light intensity, and many genes involved in defense and secondary metabolism were repressed.

### Analysis of gene expression involved in secondary metabolism and paclitaxel biosynthesis

**Fig. 6:**
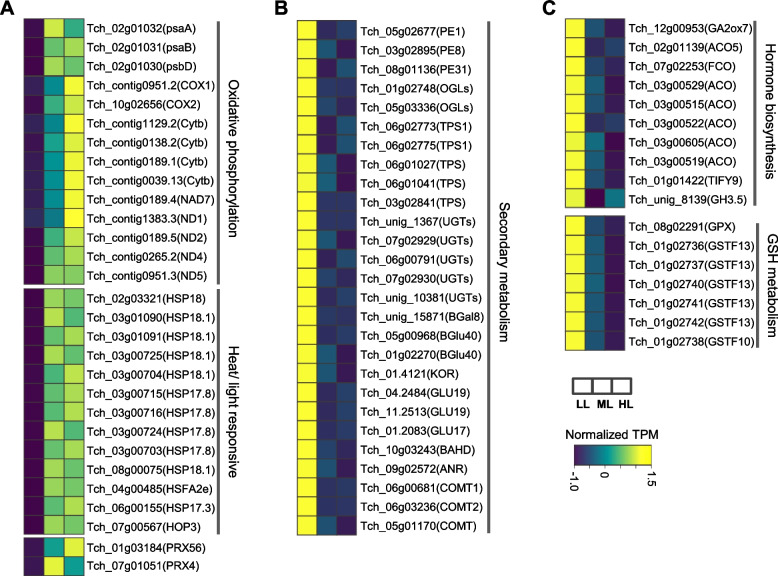
Different light intensities affect the genes expression involved in stress response and secondary metabolism in *T. chinensis*. **A** The expression patterns of the genes involved in oxidative phosphorylation and heat/light responsive, (**B**) secondary metabolism, (**C**) hormone biosynthesis, and glutathione (GSH) metabolism pathways in *T. chinensis* leaves under different light intensity treatments

We further analyzed the expression patterns of the genes enriched in specific pathways that respond to changes in light intensity. Many genes involved in oxidative phosphorylation (two cytochrome-c oxidase encoding genes [*COX*], four cytochrome-b genes [*Cytb*], five NADH dehydrogenase genes [*NDs*]), temperature- or light-responsive (heat shock proteins, *HSPs*) and oxidation–reduction processes (two peroxidase genes [*PRX*]) were upregulated in *T. chinensis* after increasing light intensity. In addition, the transcripts of three photosystem I (PSI) subunit genes (*psaA, psaB,* and *psbD*) were significantly increased in ML and HL (Fig. 6A). Numerous genes involved in multiple secondary metabolic pathways, such as terpene biosynthesis (pectinesterase [*PEs*], pectate lyase [*OGLs*], terpene synthase [*TPS*]) and phenylpropanoid biosynthesis (caffeic acid 3-O-methyltransferase genes [*COMT*], anthocyanidin reductase [*ANR*]), were downregulated by increased light intensity (Fig. 6B). Furthermore, several genes related to hormone biosynthesis (especially for ethylene), such as gibberellin-2-β-dioxygenase7 (*GA2ox7*) and 1-aminocyclopropane-1-carboxylate oxidase (*ACO*), and many glutathione metabolic genes (six GSTs and one glutathione peroxidase [*GPX*]) were significantly downregulated by increased light intensity (Fig. 6C).

**Fig. 7:**
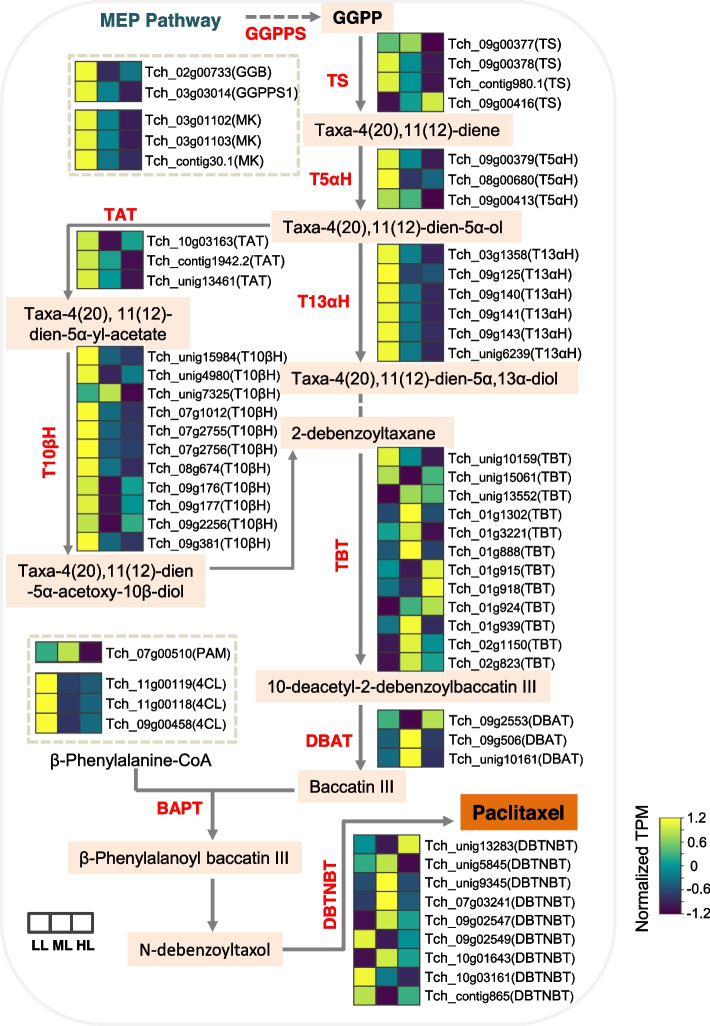
The expression patterns of the genes in paclitaxel biosynthetic pathway in *T. chinensis* under different light conditions

We analyzed the expression patterns of paclitaxel biosynthetic genes in *T. chinensis* under different light conditions. Geranylgeranyl diphosphate (GGPP) is a key precursor for paclitaxel biosynthesis and is produced by geranylgeranyl diphosphate synthase (GGPPS) in the 2-C-methyl-D-erythritol 4-phosphate (MEP) or mevalonate (MVA) pathway (Supplementary Figure 3). One GGPPS and geranylgeranyl transferase (GGB) and three mevalonate kinase (MK)-encoding genes were detected in the *T. chinensis* transcriptome, and these genes were significantly downregulated in the ML and HL groups (Fig. 7). Taxadiene synthase (TS) is a critical enzyme in paclitaxel biosynthesis. Four *TS* isoforms were identified in our data, and three were downregulated by increased light intensity. Several hydroxylases and transferases, such as taxane-5α-hydroxylase (T5αH), taxane-13α-hydroxylase (T13αH), taxane-10β-hydroxylase (T10βH), and taxadien-5α-ol-O-acetyltransferase (TAT) involved in paclitaxel biosynthesis were downregulated after increasing light intensity. Twelve taxane 2α-O-benzoyltransferase (TBT), three 10-deacetylbaccatin III 10-O-acetyltransferse (DBAT), and nine 3’-N-debenzoyl-2’-deoxytaxol-N-benzoyl transferase (DBTNBT) isoforms were identified in the transcriptome. Among them, seven *TBTs*, two *DBATs*, and five *DBTNBTs* had higher expression levels in ML than in LL and HL; two *TBTs*, one *DBAT*, and three *DBTNBTs* were downregulated in ML and HL; and the remaining isoforms (three *TBTs*, one *DBAT*, and one *DBTNBT*) had higher expression levels in HL than in ML and LL (Fig. 7). Different transferase isoforms might have distinct functional roles in paclitaxel biosynthesis and respond to environmental stimuli. In addition, we identified one phenylalanine aminomutase (PAM) and three 4-couma-rate-CoA ligase (4CL) encoding genes in the paclitaxel branching pathway. The expression of *PAM* was higher in ML than in LL and HL, but three *4CLs* were downregulated by the increased light intensity (Fig. 7). Together, our transcriptome data revealed that most paclitaxel biosynthetic genes in *T. chinensis* were repressed by increased light intensity.

**Fig. 8:**
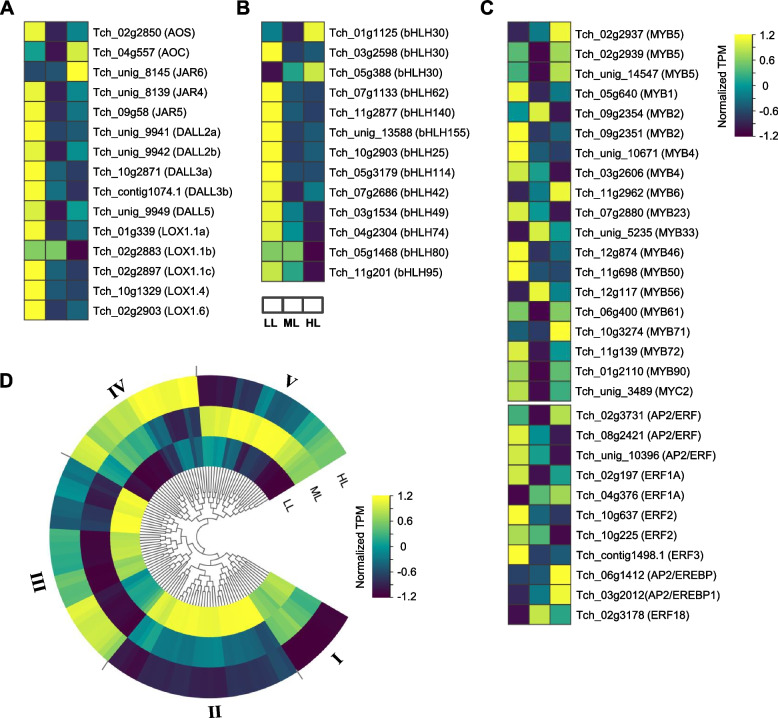
Different light conditions affect the expression of the genes and transcription factors related to paclitaxel biosynthesis in *T. chinensis*. **A** JA biosynthesis related genes, **B** the basic Helix-Loop-Helix **(**bHLH), **C** MYB and AP2/ERF families’ transcription factors, and **D** the CYP450 families’ expression patterns in *T. chinensis* leaves under different light intensity treatments

The jasmonate (JA) signaling pathway and several transcription factors, such as basic Helix-Loop-Helix (bHLH), MYB, and AP2/ERFs, are closely related to paclitaxel biosynthesis in *Taxus* [23, 26]. We analyzed the gene expression patterns of JA biosynthetic enzymes and related TFs in our transcriptome data. Allene oxide synthase (AOS) and 2 jasmonic acid-amido synthetase (JAR4 and JAR5)-encoding genes were downregulated in HL and ML, while allene oxide cyclase (AOC) and JAR6-encoding genes were upregulated in HL (Fig. 8A). JA precursor synthesis-related enzymes (DALLs and LOXs) and many bHLH genes were significantly downregulated in HL and ML (Fig. 8A, B). Some MYB (such as MYB1, MYB2b, MYB4, MYB23, MYB46 and MYB50) and AP2/ERF (ERF1A/2/3) genes were downregulated in HL or ML, while others were upregulated (such as MYB5, MYB6, MYB33 and MYB56) (Fig. 8C). The cytochrome P450 (CYP450) families play an important role in paclitaxel biosynthesis. We identified 123 CYP450 genes in our data and divided them into five classes according to their expression patterns under three light conditions. Among them, 25 CYP450s in Class II had higher expression levels in LL than in ML and HL, while 19 in Class IV and 10 in Class III had higher expression levels in HL than in ML and LL (Fig. 8D).

## Discussion

This work revealed that young *T. chinensis* saplings were very sensitive to light conditions. Exposure to high- or medium-intensity light for a long time led to oxidative stress and deleterious effects on the photosynthetic system of yew plants, including chloroplast injury, decreased chlorophyll content and photosynthetic efficiency, and photoinhibition (Fig. 1). The optimum light conditions for plants are dependent on their capacity for light energy sinking and photosynthetic activity [5]. The slow growth of yew plants may be due to their relatively low photosynthetic efficiency and biomass accumulation, especially at the sapling stage. The light responsiveness or tolerance of *Taxus* varies among different developmental stages. Previous studies have shown that large *T. baccata* plants have strong needle morphological responses to light conditions, whereas they are lacking in seedlings [9]. *Taxus* seedlings, young saplings, and juvenile plants have a lower tolerance to full sunlight than adult trees but more tolerance to shading [27–29]. Our results confirmed that yew saplings were highly susceptible to changes in light intensity.

Excessive light energy that plants cannot use to assimilate CO_2_ can be transferred to molecular oxygen, generating ROS, especially singlet oxygen (^1^O_2_), and causing photooxidative stress [5, 30, 31]. Under long-term high light, the antioxidant enzyme activities of *T. chinensis* were significantly reduced; thus, the ROS accumulated in cells that could not be scavenged and caused damage to the photosynthetic system and/or apparatus (Figs. 1 and 2). At the gene transcription level, a large number of upregulated DEGs in *T. chinensis* leaves under high or medium light were significantly enriched in response to oxidation stress and oxidation–reduction processes (Fig. 5B). High light is always combined with heat stress imposed on plants [4, 32]. In the upregulated DEGs of HL and ML, the enriched GO terms of heat stress response-related pathways were confirmed (Fig. 5B). Many heat shock protein (HSP) encoding genes were significantly upregulated in HL and ML (Fig. 6A). HSPs play a pivotal role in the reinforcement of membrane stability and detoxification of ROS and confer biotic or abiotic stress tolerance to plants [33, 34]. Therefore, these genes may play an important role in acclimation to high light and are positive for tolerance to heat stress in *T. chinensis*.

Plant cellular processes and primary/secondary metabolism are disrupted under unfavorable light conditions [4, 35, 36]. Excessive light triggers ROS generation and photooxidative stress primarily leads to lipid peroxidation and β-carotene oxidation, which further produces reactive carbonyl species (RCS) and causes H_2_O_2_ accumulation [5]. RCS may impact the conformations or functions of proteins under stress conditions, thus affecting cellular metabolic or signaling processes [37, 38]. In our data, we found that a large number of genes involved in secondary metabolism, particularly terpenoid and phenylpropanoid metabolic pathways, were significantly downregulated under HL and ML conditions (Figs. 5 and 6). As the most important secondary metabolic process, the paclitaxel biosynthesis pathway in *Taxus* was also strongly affected by light conditions. Many hydroxylase-encoding genes, such as T5αH, T13αH, and T10αH, were significantly downregulated in HL and ML (Fig. 7). Interestingly, the expression of several transferase-encoding genes, such as *TBT, DBAT*, and *DBTNBT*, was induced by medium light but depressed by high light. These genes may have a function in response to environmental stimuli, and their functional mechanisms should be further studied.

It is proposed that JA, as a possible signal in systemic acquired acclimatization (SAA), is induced by high light in plants [39]. However, plants exposed to high light for a long time produce ROS, thus affecting JA biosynthesis or signaling. It has been suggested that the JA signaling pathway is closely related to paclitaxel biosynthesis in *Taxus* [23, 26, 40]. Xiong et al. reported that JA treatment could promote CYP450 gene expression, which was related to paclitaxel biosynthesis and could enhance the baccatin III and paclitaxel content in the *Taxus* cell line [23]. Majeed et al. showed that JA biosynthesis and taxol production were correlated in *T. contorta* in different seasons. Our results showed that JA signaling and paclitaxel biosynthetic genes were commonly downregulated under increased light intensity, which was highly consistent with previous studies. The CYP450 families participate in nearly half of the enzymatic reactions in paclitaxel biosynthesis in *Taxus* [23, 41]. In our study, we found different expression patterns of CYP450 in *T. chinensis* leaves under different light conditions (Fig. 8D). Several genes were highly expressed in HL that possibly play a positive role in tolerance to high light or heat stress. Furthermore, some TFs, including bHLH, MYB, and AP2/ERF, were related to paclitaxel biosynthesis [23, 26]. In our data, many bHLH genes were downregulated under HL or ML conditions, while some MYB and AP2/ERF genes were induced by HL or ML. These TFs may paly vital roles in phototolerance in *Taxus* and are worth studying in the future.

Understanding how environmental factors influence the growth and development of *Taxus* is essential for the protection and recovery of endangered species by artificial regeneration or cultivation. Light, as the major environmental factor, is critical for the growth and development of yew trees. Our work investigated the physiological and transcriptomic responses to different light conditions in *T. chinensis* saplings and revealed that photosynthesis, cellular metabolism, and paclitaxel biosynthesis were affected by increased light intensity. We identified several TFs, such as HSP, MYB, AP2/ERF and CYP450s, that were induced by high or medium light. These genes may paly important roles in acclimation to excess light or heat stress in *Taxus*, and their functions need to be further studied.

## Conclusions

In summary, this study combined physiological, biochemical, and transcriptomic methods to analyze the *T. chinensis* response to different light conditions and revealed that young yew saplings were prone to being influenced by changes in light conditions. The increased light intensity severely impacted the photosynthetic system and caused photooxidative stress in *T. chinensis* saplings. High or medium light also disrupts secondary metabolic processes and suppresses paclitaxel biosynthesis. A large number of genes related to paclitaxel biosynthesis were downregulated after the light intensity increased. Moreover, we identified several paclitaxel biosynthetic transferase-, MYB-, AP2/ERF-encoding genes and CYP450s that were induced by increased light and inferred that these genes may play important roles in phototolerance or heat stress in *Taxus*.

## Materials and Methods

### Plant material and light treatments

*Taxus chinensis* (Pilger) Rehd. seeds were collected from the natural habitat (105.73°N, 33.74°E) in Chengxian County, Gansu Province, Northwest China. Permission to collect the seeds of *T. chinensis* was provided by the Yuhe National Nature Reserve, Gansu Province. The formal identification of the plant material was conducted by Prof. Fengling Yang. The seeds were germinated in a greenhouse in 2016. In spring, 5-year-old healthy *T. chinensis* seedlings with uniform size were transplanted into 10L pots filled with homogenized soil and grown in a natural lit glass greenhouse under shading condition (light intensity about 7000 lx), with a temperature range of 24℃ ~ 30℃ and relative humidity of 75%. All the pots regularly watered with 1/2 Hoagland solution. After 2 months, 54 plants with similar height (~ 50 cm) were selected for the experimental treatments. All the plants were divided into three groups for different light intensity treatments (each treatment with three biological replicates and six plants for per replicate): (1) high light (HL), with 13,000 lx illuminance, which was similar to the full sunlight at natural habitat, (2) medium light (ML), with 10,000 lx illuminance, which was similar to the half shading condition, (3) low light (LL), with 7000 lx illuminance, which was similar to the shading condition. Plants were exposed to light for 12 h/day (from 08:00 to 20:00) from full spectrum LED light lamps (HSQ-ZPJ-CC, Beijing Hongshangqi, Co., Ltd, China) and placed 100 cm above the plants. The light intensity was measured with a LX1010 digital illuminometer (Beijing Normal University, China). After 4 weeks, the fully expanded leaves of the three treatment group plants were sampled for measure the physiological and biochemical indices and for RNA sequencing, each sample with three biological replicates. The collected leaves were immediately placed in liquid N_2_ and stored at –80 °C for further experiments. The voucher specimen has been deposited in the herbarium of Henan University of Urban Construction (Deposition number not available). Our experimental research of the *Tauxs* trees complied with local legislation, national and international guidelines.

### Measurement of chlorophyll contents and chlorophyll fluorescence

Chlorophyll contents were determined according to the method as described previously [42]. In brief, leaves of *T. chinensis* were weighed (0.2 g, fresh weight) and homogenized in 4 mL of 80% acetone for chlorophyll extraction. The contents of chlorophyll *a* (Chl *a*) and Chl *b* were measured using a spectrophotometer at absorbances of 662 nm and 644 nm, respectively. Chl *a* fluorescence was measured at 9:00 ~ 11:00 a.m. using a portable PAM-2500 chlorophyll fluorometer (Walz, Eichenring, Germany) on the leaves near the apex of *T. chinensis* plants. The leaves were dark-adapted for 20 min, a PAR of 900 μmol m^−2^ s^−1^ was used for the measurement.

### Transmission Electron Microscopy (TEM) observations

TEM was performed on a section (1 ~ 2 mm in length) of a fully expanded leaf near the apex of *T. chinensis* plants (HL, ML and LL conditions) to observe chloroplast ultrastructure as described previously [43]. Leaf sections were fixed with 3% glutaraldehyde (v/v) in 0.1 M phosphate buffer (pH 7.2) for 6 h at 4 °C, followed by 2 h of post-fixation in 1% osmium tetraoxide. Samples were rinsed three times with phosphate buffer (0.1 M, pH 7.2), dehydrated in a graded ethanol series (50, 60, 70, 80, 90, 95, and 100%) and embedded in eponaraldite. Ultrathin Sects. (80 nm) were sliced, stained with uranyl acetate and lead citrate, and mounted on copper grids for viewing using an H-600IV TEM (Hitachi, Tokyo, Japan).

### Determinations of antioxidant enzyme activities, Proline and soluble sugar contents

Proline concentration was measured as described previously [36]. In brief, 0.5 g fresh leaves were homogenized in 5 mL of 3% sulfosalicylic acid solution. After centrifugation, 2 mL of supernatant, 2 mL of glacial acetic acid, and 2 mL of 2.5% acid ninhydrin solution were added to a tube and covered with Teflon cap. Absorbance of the free proline was measured at 520 nm using a UV/visible spectrophotometer (GENESYS™ 10S, Thermo Scientific, USA). The soluble sugar content was determined according to the method as described previously [44]. For antioxidant enzymes activities assay, 0.5 g fresh leaves were ground in liquid nitrogen and extracted with 50 mM potassium phosphate buffer (pH 7.8) containing 0.1 mM EDTA, 1% (w/v) polyvinyl pyrrolidone (PVP), 0.1 mM phenylmethane sulfonyl fluoride (PMSF) solution and 0.2% (v/v) Triton X-100. Superoxide dismutase (SOD, EC 1.15.1.1) activity was assayed by monitoring the inhibition of photochemical reduction of nitro-blue tetrazolium (NBT) as described previously [45]. The peroxidase (POD; EC1.11.1.7.) activity was measured at 470 nm, as described previously [46]. Catalase (CAT; EC 1.11.1.6.) activity was assayed as described previously [47].

### Determinations of paclitaxel content

The paclitaxel (taxol) content in *T. chinensis* leaves was measured by ultra-performance liquid chromatography-tandem mass spectroscopy (UPLC-MS/MS) system (Agilent, CA, USA). For taxol extraction, 1.0 g leaves (dry weight, DW) of *T. chinensis* were homogenized in 20 mL of 80% ethanol solution (ethanol: ddH_2_O, v/v) by a homogenizer for 3 min. After centrifugation at 1,776 g for 5 min at 4 °C, transfer the supernatant to a new tube. Add 10 mL of 80% ethanol solution to the sediment and sonicated for 30 min using an ultrasonic processor, then centrifugation for 5 min and transfer the supernatant, repeat the step for 3 times. Combined all the extracts and dried under nitrogen. Add 2 mL of 80% methanol solution to resuspended the dried extracts and filtered with a 0.45 μm microporous membrane (Entegris| ANOW, China) for UPLC test. UPLC separation of taxol from *T. chinensis* leaves with a 5 μm (250 mm × 4.6 mm) Hypersil ODS C18 column (Thermo Scientific, USA). The mobile phase consisted of 35% of solvent A (2 mM ammonium formate and 0.1% formic acid aqueous solution) and 65% of solvent B (100% methanol). The flow rate was 1.0 mL min^−1^ and the column oven temperature was maintained at 25 °C for the duration of analysis. The injection volume for each sample was 10 μL. The samples were detected with a diode array detector set at 227 nm, each sample with three replicates.

### RNA extraction, library construction, and mRNA sequencing

Total RNA was isolated from *T. chinensis* leaves using TRIzol reagent (Thermo Scientific, USA) according to the manufacturer’s protocol. Each treatment sample had three biological replicates. RNA concentration and purity was measured using a Qubit 2.0 Fluorometer (Invitrogen, USA). RNA integrity was measured using an Agilent Bioanalyzer 2100 (Agilent Technologies, USA). High-quality RNA was processed for RNA-seq library construction. A total of 2 µg RNA was used for mRNA isolation, mRNA fragmentation and cDNA library construction were conducted using a NEBNext®Ultra™ RNA Library Prep Kit (NEB, USA) according to the manufacturer’s protocol. The index codes were added to attribute sequences to each sample. The cDNA libraries were sequenced at Biomarker Technologies (Beijing, China) on the Illumina NovaSeq 6000 System by 150 bp paired-end sequencing.

### Analysis of RNA-seq and identification of differentially expressed genes (DEGs)

RNA-seq raw reads were filtered to remove adapter sequence and low-quality reads by Trimmomatic (v0.36) software. The clean reads were aligned to the *T. chinensis* reference genome which published recently [23] using HISAT2 (v2.1.0) software. Gene expression levels were quantified by the R package DESeq2 (v1.6.3) with parameters for strand-specific RNA-seq [48]. Differentially expressed genes (DEGs) were identified between two comparisons using the following criteria: |log2 (fold change)|> 1 and false discovery rate (FDR) < 0.01. The FDR was generated from an adjusted P-value using the Benjamini–Hochberg method. Gene function annotations were performed by alignment the gene sequence to NCBI non-redundant protein sequences (NR, ftp://ftp.ncbi.nih.gov/blast/db/), Swiss-Prot [49], Gene Ontology (GO) [50], Kyoto Encyclopedia of Genes and Genome (KEGG) [51], Protein family (Pfam) [52] and Clusters of Orthologous Groups (COG) [53] databases using BLASTX with a significance threshold of E ≤ 1.0 × 10^–5^. Blast2GO (v2.5) software was used for enrichment of the GO terms based on the Nr annotation, and KEGG database was used to determine metabolic pathways of the genes. Heatmaps were generated using TBtools (v1.086) software [54].

### Statistical analysis

Each set of data were separately analyzed using SPSS software (v19.0). Each bar represents the mean ± *SE* of at least three replicates. Different letters above the bars indicate significant differences, and values of *P* < 0.05 represented statistical significance using *Tukey’s* test.

## Supplementary Information

**Additional file 1.** 

## Data Availability

The RNA-seq data generated in this study were deposited in the NCBI Sequence Read Archive (BioProject ID: PRJNA848951; https://submit.ncbi.nlm.nih.gov/subs/sra/). The datasets supporting the conclusions of this article are included within the article and its additional files.

## Abbreviations

APX: Ascorbate peroxidase
BAPT: Baccatin III-13-O-phenylpropanoyl transferase
CAT: Catalase
COG: Clusters of Orthologous Groups
DBAT: 10-Deacetylbaccatin III 10-O-acetyltransferse
DBTNBT: 3’-N-debenzoyl-2’-deoxytaxol-N-benzoyl transferase
DEGs: Differentially expressed genes
FDR: False discovery rate
GGPP: Geranylgeranyl diphosphate
GO: Gene Ontology
GST: Glutathione S-transferase
HL: High light
KEGG: Kyoto Encyclopedia of Genes and Genomes
LL: Low light
MEP: 2-C-methyl-D-erythritol 4-phosphate
ML: Medium light
MVA: Mevalonate
PAM: Phenylalanine aminomutase
PCA: Principal component analysis
POD: Peroxidase
ROS: Reactive oxygen species
SOD: Superoxide dismutase
TAT: Taxadien-5α-ol-O-acetyltransferase
TBT: Taxane 2α-O-benzoyltransferase
T2αH: Taxane-2α-hydroxylase
T5αH: Taxane-5α-hydroxylase
T7βH: Taxane-7β- hydroxylase
T10βH: Taxane-10β-hydroxylase
T13αH: Taxane-13α-hydroxylase
TS: Taxadiene synthase
UPLC-MS/MS: Ultra-performance liquid chromatography-tandem mass spectroscopy
