# Supplementary material for: Transcriptome analysis provides insights into light condition effect on paclitaxel biosynthesis in yew saplings
Source: BMC Plant Biol. 2022 Dec 12;22:577. doi: 10.1186/s12870-022-03958-2 (PMC9743728; doi:10.1186/s12870-022-03958-2)
Supplement: Supplementary file 1 — Additional file 1. [file 12870_2022_3958_MOESM1_ESM.pdf]

Supplementary Figures

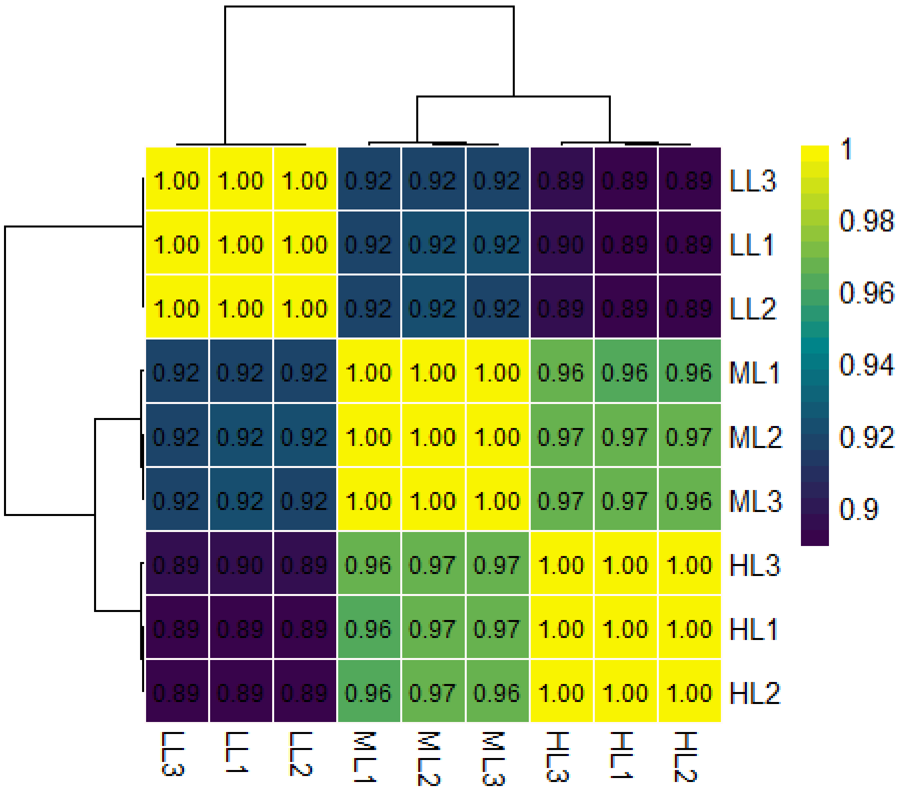

Supplementary Fig. 1. Heatmap and Pearson correlation coefficients for RNA-seq replicates of *T. chinensis* under different light conditions. HL, high light; ML, medium light; LL, low light. Three biological replicates are shown.

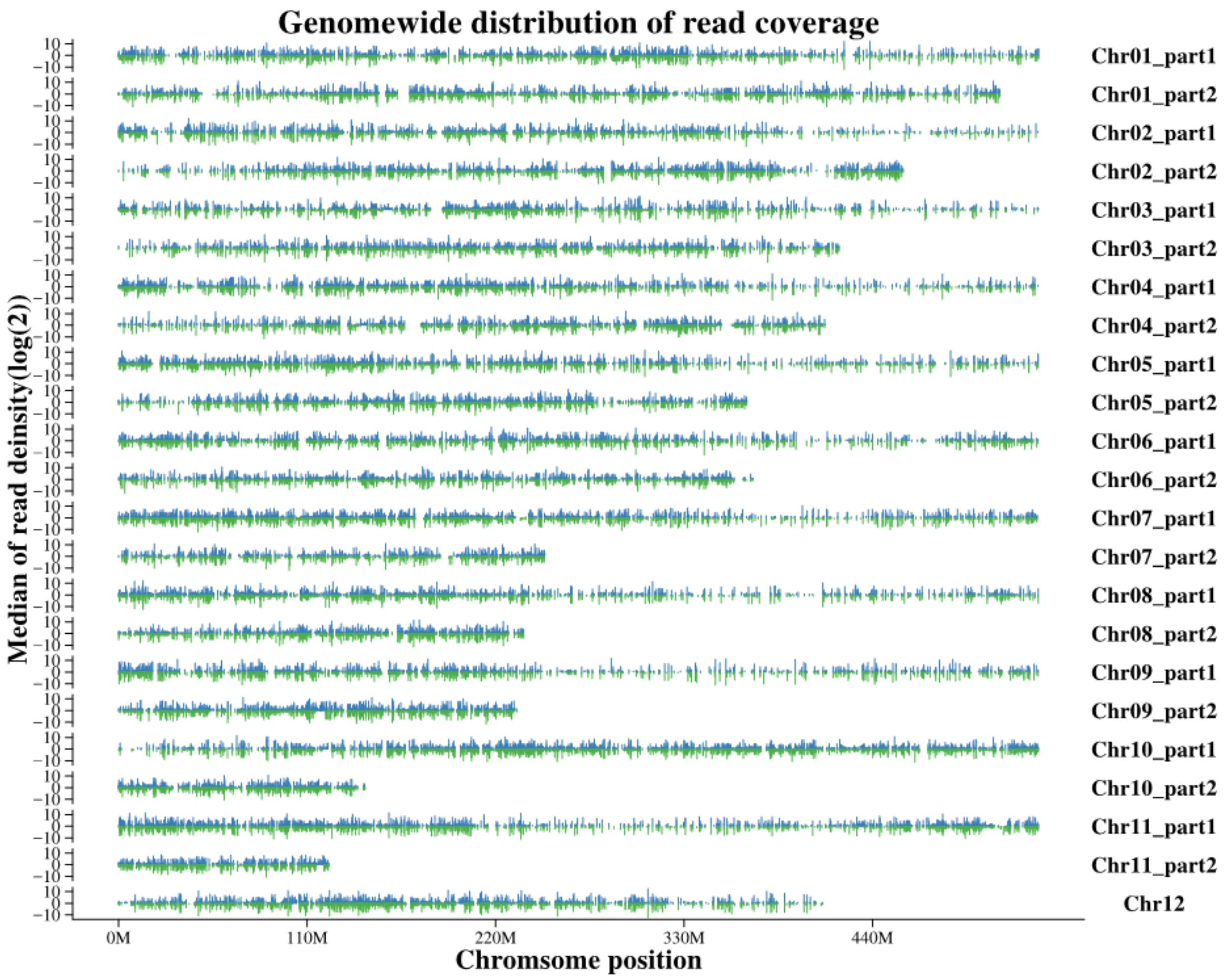

Supplementary Fig. 2. The distribution and coverage of the mapped reads on the reference genome of *T. chinensis*.

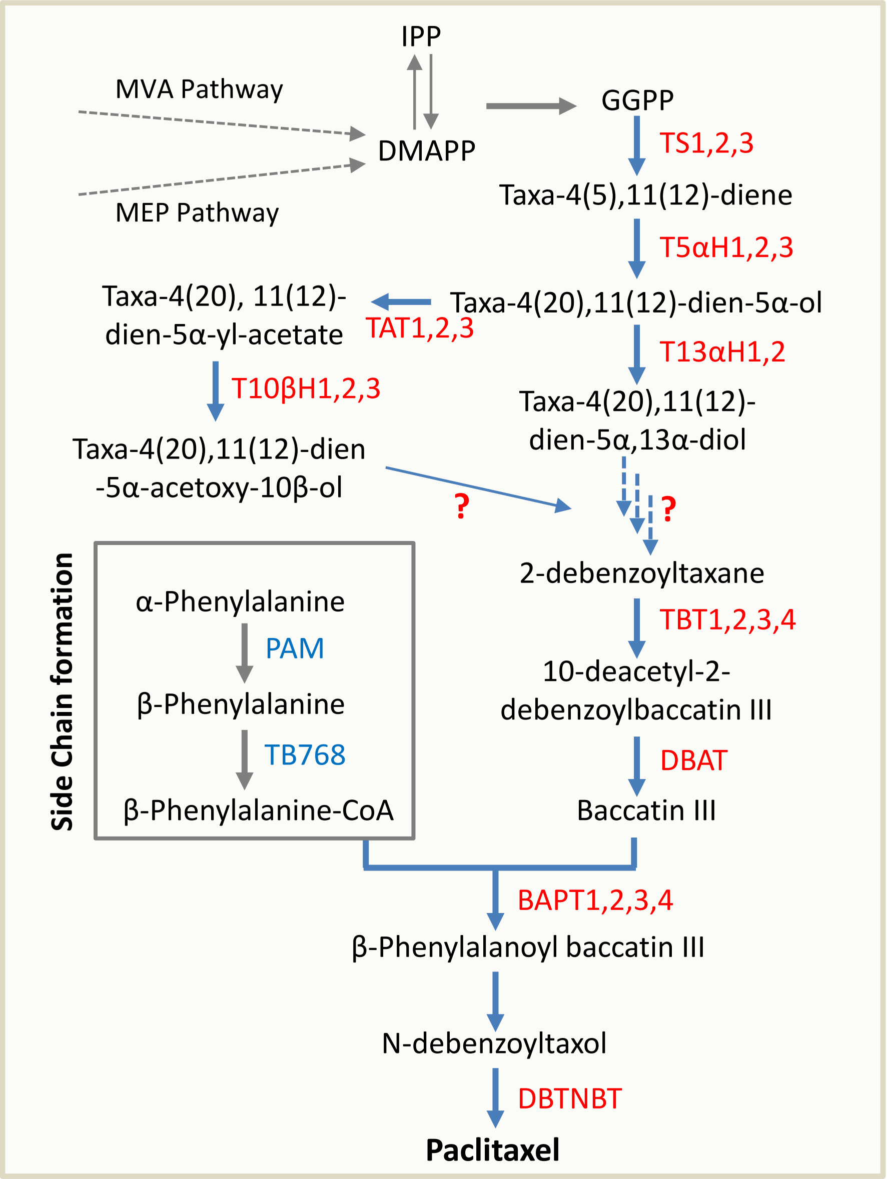

Supplementary Fig. 3. The paclitaxel biosynthesis pathway.
